# Social stigmatisation among COVID-19 patients: addressing a potential source of COVID-19 transmission to healthcare staff in cardiac emergency and cardiac care units

**DOI:** 10.1192/bji.2020.44

**Published:** 2020-08-24

**Authors:** Farhan Ali, Sheikh Muhammad Ebad Ali

**Affiliations:** 1 MBBS, House Officer, Dr Ruth KM Pfau Civil Hospital Karachi, Karachi, Pakistan; 2 MBBS, House Officer, Dr Ruth KM Pfau Civil Hospital Karachi, Karachi, Pakistan. Email sheikh.muhammadebadali14@dmc.duhs.edu.pk

**Keywords:** Low and middle income countries, social deprivation, stigma and discrimination, psychosocial interventions, social functioning

## Abstract

During our routine work, we noticed an increased incidence of COVID-19 diagnoses among patients in the cardiac unit, which led to an exponential increase in COVID-19 cases among hospital staff. We found that patients hid their symptoms from the emergency doctors and attributed those symptoms to cardiac or other causes. Social stigmatisation appeared to be the root cause for hiding their symptoms. Hence, we recommended a strategy to introduce psychological counselling of patients who were suspected to be infected with COVID-19, with a normal cardiac workup to overcome social stigmatisation and save our general wards from COVID-19.

The first case of severe acute respiratory syndrome coronavirus 2 (SARS-CoV-2) appeared in December 2019 from Wuhan Province, China.^1^ This virus has now spread to 213 countries and territories around the world.^2^ Because of its high rate of transmission from person to person and high rate of morbidity, there is fear in society and it has been reported that individuals avoid contact with anyone they think might have COVID-19.^3^ Such public anxiety and fear leads to psychosocial hurdles such as social stigma and discrimination.^3,4^ These challenges mean that healthcare professionals are dealing with patients who attribute their symptoms to cardiological problems while hiding the symptoms of COVID-19 because of social stigma.^5^

## Identifying the problem

We are currently posted in a 1900-bed tertiary care hospital in Karachi, Pakistan, where a 350-bed COVID-19 isolation unit and intensive care unit (ICU) have been set up. During the 4 months after the first case of COVID-19 was reported in Pakistan (on 26 February 2020), a wave of fear developed across the country. Awareness campaigns regarding the symptoms of COVID-19 were launched by the government on television and in the print media. The major objective of those campaigns was to decrease the number of COVID-19 cases by educating the population. However, a new trend was reported while we were working in the COVID-19 isolation unit and intensive care unit (ICU). A higher than routine number of patients were being transferred from the cardiac emergency unit and the cardiac care unit (CCU) to the COVID-19 isolation unit and ICU. It was seen that patients who had a cough, fever or shortness of breath were hiding their symptoms to avoid discrimination and, on inquiry, they believed that they would not have been treated by healthcare workers if they had revealed their symptoms. This led to a higher incidence of healthcare staff being tested positive for SARS-CoV-2 in the cardiac emergency unit and CCU.

## Intervention

This misbelief, which prevails in society and seems to be the reason behind the misconception of discrimination, is a major hurdle faced by healthcare workers in cardiac emergency units and CCUs. This social reaction causes difficulty in the diagnosis of patients who have COVID-19 symptoms and may have a detrimental effect on efforts to limit the spread of the disease, as well as putting the lives of healthcare workers in non-COVID-19 critical care units, ICUs and CCUs in danger.^2^ Our observations led us to the conclusion that we should focus on stigmatisation along with the treatment of COVID-19. Hence, we prospectively started a 2 min counselling session for all patients who were attributing their symptoms to cardiac disease without any sound findings on cardiac investigations in the cardiac emergency unit. The counselling points were designed after a detailed discussion with a psychologist and psychiatrist. The counselling session addressed the following issues:

- building a rapport with the patient
- asking them about their knowledge and beliefs regarding COVID-19
- correcting their misunderstandings
- a gesture of sympathy and empathy, to release their fear and anxiety before asking COVID-19-related questions.

**Fig. 1:**
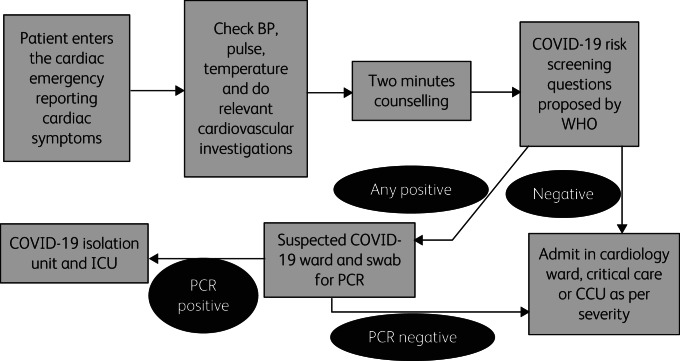
Flowchart for admission of patients to a cardiology ward, critical care unit, cardiac care unit (CCU) or isolation unit and intensive care unit (ICU). BP, blood pressure; WHO, World Health Organization; PCR, polymerase chain reaction test.

We noticed a change in the previously observed trend, and a significant drop in the referral of patients from the CCU and critical care was reported. We are also pleased to report that, after adding the 2 min counselling sessions, no nursing staff were reported positive for SARS-CoV-2 in the CCU. We would like to propose our strategy as a flowchart shown in Fig. 1.

## Conclusions

Healthcare workers must deal with the social stigma surrounding COVID-19 infection, to reduce the fear, worry and anxiety of patients who have symptoms. Counselling can play an important role in dealing with the social reaction. Healthcare workers need to counsel patients, to lessen their anxiety so that they become more comfortable revealing their symptoms. This can help the diagnosis, treatment and quarantining of patients who have COVID-19. The counselling may also be fruitful in reducing the incidence of SARS-CoV-2 transmission to nurses and doctors working in cardiac emergency units and CCUs, by allowing a timely diagnosis. Public health education can also play a major role in reducing stigmatisation and the cultural impact of the pandemic in the general population, thus reducing the number of COVID-19 cases, morbidity and mortality.^6^

## References

[1] Huang C, Wang Y, Li X, Ren L, Zhao J, Hu Y, Clinical features of patients infected with 2019 novel coronavirus in Wuhan, China. Lancet 2020; 395: 497–506. 31986264 10.1016/S0140-6736(20)30183-5 PMC7159299

[2] Worlodometer. Countries where COVID-19 has spread. Worlodometer, 2020 (https://www.worldometers.info/coronavirus/countries-where-coronavirus-has-spread/ [cited 1 Aug 2020]).

[3] Lin C-Y. Social reaction toward the 2019 novel coronavirus (COVID-19). Soc Health Behav 2020; 3: 1.

[4] Taylor S, Landry CA, Paluszek MM, Fergus TA, McKay D, Asmundson GJG. Development and initial validation of the COVID Stress Scales. J Anxiety Disord 2020; 72: 102232. 32408047 10.1016/j.janxdis.2020.102232 PMC7198206

[5] Ahorsu DK, Lin CY, Imani V, Saffari M, Griffiths MD, Pakpour AH. The fear of COVID-19 Scale: development and initial validation. Int J Ment Health Addict [Epub ahead of print] 27 Mar 2020 Available from: 10.1007/s11469-020-00270-8. PMC7100496 32226353

[6] Bruns DP, Kraguljac NV, Bruns TR. COVID-19: facts, cultural considerations, and risk of stigmatization. J Transcult Nurs 2020; 31: 326–32. 32316872 10.1177/1043659620917724 PMC7324134

